# Boosting furfural production by combining polyoxometalates and ionic liquids for effective fractionation of lignocellulosic biomass†

**DOI:** 10.1039/d5ra02401c

**Published:** 2025-05-02

**Authors:** Max P. Papajewski, Suhaib Nisar, Chaoyue Zhang, Jason P. Hallett, Jakob Albert

**Affiliations:** a Institute for Technical and Macromolecular Chemistry, Universität Hamburg Bundesstraße 45 20146 Hamburg Germany jakob.albert@uni-hamburg.de; b Department of Chemical Engineering, Imperial College London, South Kensington Campus London UK

## Abstract

This study investigates the use of ionic liquids (ILs) and polyoxometalates (POMs) to enhance furfural production from lignocellulosic biomass potentially under milder conditions. The optimized reaction parameters achieved up to 75% yield, with sugarcane bagasse providing the highest efficiency, demonstrating a promising pathway for sustainable chemical production.

## Abstract 2

This study investigates the use of ionic liquids (ILs) and polyoxometalates (POMs) to enhance furfural production from lignocellulosic biomass potentially under milder conditions.

## Graphical abstract

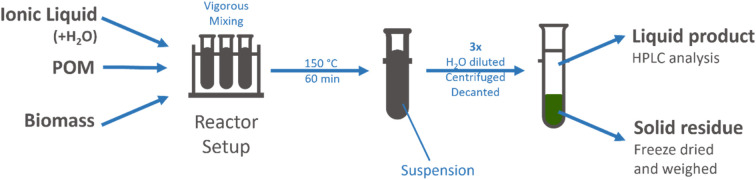

Climate change is becoming an increasingly urgent global issue, necessitating a significant reduction in greenhouse gas emissions to mitigate further global warming. Among these gases, carbon dioxide (CO_2_) is the most impactful, primarily generated through the combustion of fossil fuels such as coal, crude oil, and natural gas.^1^ Additionally, a substantial portion of carbon-based pharmaceuticals and industrial chemicals is derived from crude oil, further reinforcing its dependence and continued usage.^2^

To ensure sustainability, viable alternatives to crude oil must be identified. While alternative energy sources, such as electric and hydrogen-powered vehicles, are already commercially available for fuel applications, no widespread replacement exists for the production of most pharmaceuticals and industrial chemicals.^3–5^ One promising renewable feedstock in this regard is lignocellulosic biomass. As it is derived from atmospheric CO_2_ through photosynthesis, it offers the dual advantage of being inherently carbon-based while also enabling the production of CO_2_-neutral downstream products.^6–8^

For certain specialty chemicals, the use of lignocellulosic biomass as a feedstock is already well-established, as exemplified by furfural production. Furfural is a versatile platform chemical with applications such as extracting agent in petroleum refining, as a fuel additive, and as an intermediate in various industries, including resin production, agriculture (as an herbicide or pesticide), and the food sector.^9,10^ Beyond its diverse applications, furfural's production pathway is particularly relevant in the context of defossilization. With a capacity of 400 kta and China as a main producer, the production relies almost exclusively on hemicellulose-rich byproduct streams such as corncobs and sugarcane bagasse.^11,12^ As these feedstocks are derived from atmospheric CO_2_, furfural inherently benefits from a carbon-neutral supply chain. However, the dominant industrial production method, the Quaker Oats process, operates under harsh conditions, employing high temperatures (160–190 °C) and sulfuric acid as a hazardous and non-sustainable hydrolysis catalyst. Despite these intensive conditions, furfural yields based on the pentosan content typically do not exceed 50%. Furthermore, the remaining biomass constituents, primarily cellulose and lignin, undergo extensive degradation, leading to significant non-usable waste.^9,13–15^

The limitations of the Quaker Oats process for furfural production present an opportunity to develop alternative routes under milder conditions while preserving cellulose and lignin. Ionic liquids (ILs) have been extensively studied as a solution, serving as both a fractionating solvent and a Brønsted acidic catalyst to enhance furfural yields and to enable lignin isolation.^16–21^ For example, certain ILs have achieved furfural yields of up to 81% under optimized conditions.^22^ Building on this concept, recent research has explored the use of polyoxometalates (POMs) in combination with ionic liquids, which offer the potential to further improve furfural yield and selectivity under milder conditions, reducing cellulose degradation.^23–27^ Bukowski *et al.* introduced a Brønsted acidic polyoxometalate (POM), which serves as a starting point for further improving furfural production efficiency.^23,24^

The objective of this study was to identify a suitable combination of IL and POM that enables high furfural yields under mild reaction conditions (≤150 °C) avoiding carbon loss and waste production. Sugarcane bagasse was selected as the model xylan-rich biomass feedstock among others (Table S2†), and a range of both commercially available and synthesized ILs and POMs were evaluated (sources and synthesis details are provided in the ESI, Table S1 and Fig. S1†).

Experiments were conducted in pressure-resistant, sealable glass tubes (Fig. S2 and S3†). The solid components (feedstock and POM) were weighed first, followed by the addition of the solvent (IL and deionized water) in proportion to their respective masses. The tubes were then sealed and mixed thoroughly using a vortex mixer, ensuring no solids adhered to the tube walls. The sealed tubes were secured in a rack and placed into a preheated oven at the designated reaction temperature. The reaction time commenced immediately upon heating and was terminated by transferring the rack to a fume hood for cooling.

Once the samples reached room temperature, deionized water was added to precipitate lignin. The solids and liquids were then separated by centrifugation and decantation, with the liquid phase masses being recorded. Furfural concentration in the resulting liquid product was quantified using high-performance liquid chromatography (HPLC).

For the initial screening of ILs, silicotungstic acid (H_4_SiW_12_O_40_, HSiW) was chosen as the standard POM. The experiments were carried out at 150 °C with a reaction time of 60 minutes, using 0.25 g sugarcane bagasse, 5 g aqueous ionic liquid (including 20 wt% deionized water), and 0.2 g POM. The results are summarized in Table 1.

**Table 1:** Furfural yields for initial ionic liquid screening combined with silicotungstic acid (HSiW). Reaction conditions: 0.25 g sugarcane bagasse, 5 g ionic liquid (20 wt% H_2_O), 0.20 g H_4_SiW_12_O_40_ (HSiW), *t* = 60 min, *T* = 150 °C

Entry	Ionic liquid	Polyoxometalate	Furfural yield (%)
1	[Hmim][Cl]	HSiW	46.8
2	[Bmim][Cl]	HSiW	40.8
3	[Emim][Cl]	HSiW	40.6
4	[Emim][Et2PO_4_]	HSiW	2.8
5	[TBMP][MeSO_4_]	HSiW	42.0
6	[DMBA][HSO_4_]	HSiW	61.2
7	[DMBA][HSO_4_]	—	55.8

With the exception of entry 4, all IL + POM systems achieved notable furfural yields ranging from 40.6% to 61.2%. A comparison between entries 3 and 4, both of which utilize the [Emim] cation, reveals that the [Et_2_PO_4_] anion has a substantial negative impact on furfural yield, reducing it to just 2.8%. Additionally, after the reaction, the liquid from entry 4 exhibited a pronounced change of colour, whereas all other samples were slightly yellow. This suggests an undesired chemical reaction between [Emim][Et_2_PO_4_] and HSiW leading to an unstable catalytic system reducing the furfural yield. Among the tested ILs, entry 6 ([DMBA][HSO_4_] + HSiW) demonstrated the highest furfural yield of 61.2%, significantly outperforming the other systems. This is likely due to the protic nature of the hydrogen sulfate anion, as Brønsted acidity is known to promote the dehydration of xylose to furfural as well as its balanced acid to base ratio (Table S3†).^16,28,29^ Furthermore, a comparison between entries 6 and 7 highlights the beneficial role of the POM, which increased the furfural yield by approximately 5%, emphasizing its catalytic contribution to the process.

Next, the influence of different POMs on the furfural yield was investigated. The experimental procedure remained consistent with the previous experiments, maintaining fixed POM masses. The tested POMs included H_3_PMo_12_O_40_ (HPMo), H_4_SiMo_12_O_40_ (HSiMo), H_3_PW_12_O_40_ (HPW), and H_4_SiW_12_O_40_ (HSiW), with the results summarized in Table 2.

**Table 2:** Influence of various POMs on furfural yield. Reaction conditions: 0.25 g sugarcane bagasse, 5 g IL (20 wt% H_2_O), 0.20 g POM, *t* = 60 min, *T* = 150 °C

Entry	Ionic liquid	Polyoxometalate	Furfural yield (%)
1	[DMBA][HSO_4_]	HPMo	49.1
2	[DMBA][HSO_4_]	HSiMo	38.0
3	[DMBA][HSO_4_]	HPW	56.4
4	[DMBA][HSO_4_]	HSiW	61.2
5	[DMBA][HSO_4_]	—	55.8

With the exception of entry 2, the data reveal a clear trend of increasing furfural yield with increasing size POM's constituents. This behaviour can be attributed to the intrinsic differences in the atomic properties of tungsten and molybdenum. Tungsten atoms are larger and more polarizable than molybdenum, which results in tungsten-based polyoxometalates (POMs) generally exhibiting larger overall structures and enhanced electronic delocalization. These characteristics contribute to improved stabilization of charge separation upon proton dissociation, potentially leading to a higher degree of proton release into the medium enhancing catalytic activity.

However, HSiMo in entry 2 and HPMo in entry 1 deviate from this pattern, exhibiting the lowest furfural yields (38.0% and 49.1%, respectively). A possible explanation is a potential instability of phosphomolybdate POMs under the applied reaction conditions. HSiMo was synthesized in the lab and displayed temperature-sensitive behaviour, which may have led to its degradation at elevated reaction temperatures.

The chemical system was subsequently optimized using a design of experiments (DoE) approach,^30^ specifically a Box–Behnken design,^31^ to investigate the effects of temperature, reaction time, water loading, and catalyst loading. The following variables have been varied:

• Temperature: 110, 130, 150 °C.

• Reaction time: 30, 60, 90 min.

• Water loading: 20, 40, 60 wt%.

• Catalyst loading: 1, 2.5, 4 wt%.

A full-factorial, three-level design of experiments study was performed (Table S5†). Water and catalyst loadings were based on the overall mass of solvent. The furfural yields of all 27 individual experiments are summarized in Table S5,† and the model was validated using Statistica™ software. The effects of the reaction parameters are shown in Fig. 1.

**Fig. 1:**
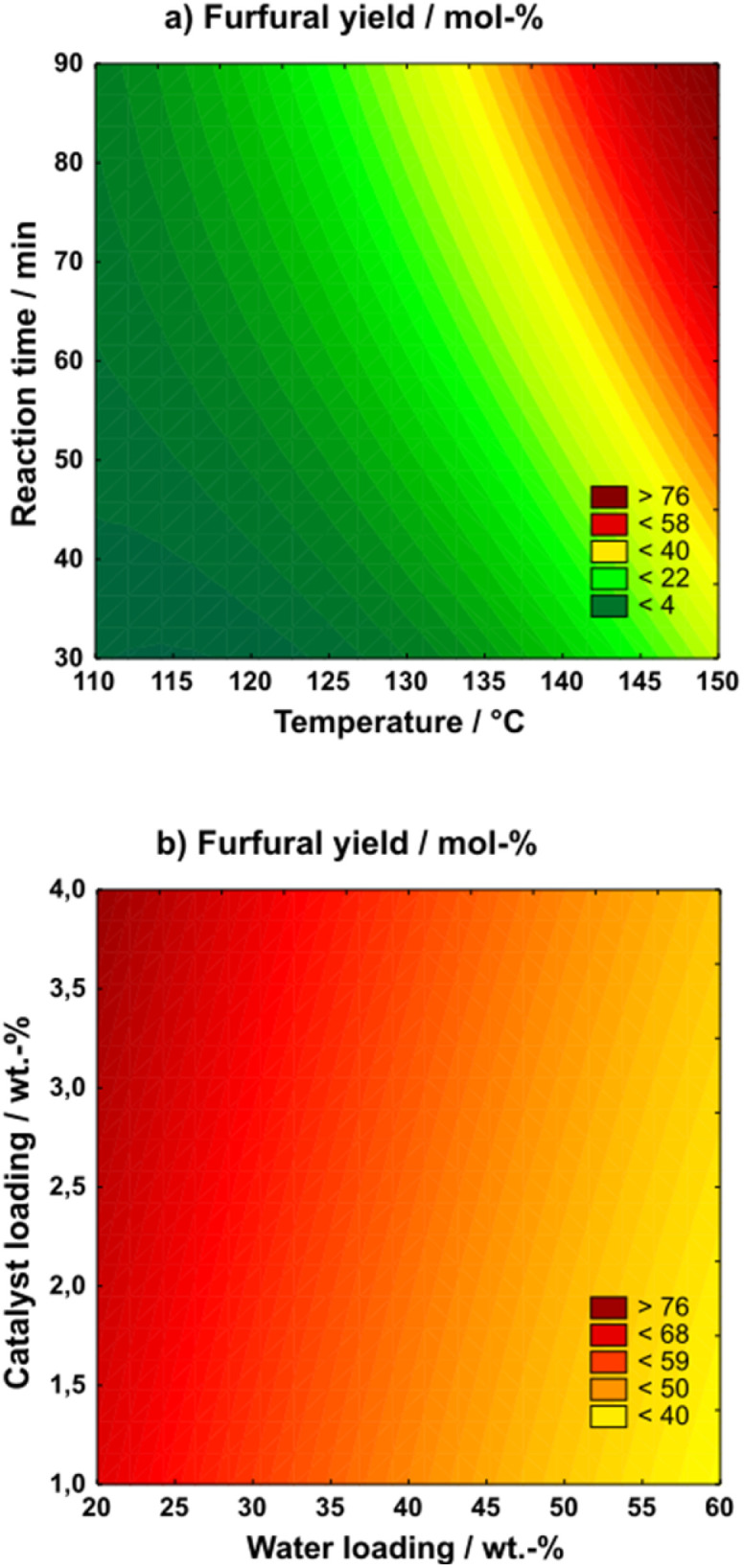
DoE study for optimizing furfural yield depending on the relationship of reaction time *vs.* temperature (a) and catalyst loading *vs.* water loading (b). Reaction conditions: 0.25 g substrate, 5 g [DMBA][HSO_4_] including deionized water, H_4_SiW_12_O_40_ (HSiW).

As seen, the reaction temperature and time have the greatest impact on furfural yield, also depicted by the Pareto chart in Fig. S4.† The highest yields, approximately 75%, were achieved at 150 °C and 90 minutes. Below a temperature of around 120 °C or reaction times shorter than 30 minutes, furfural yields became negligible, indicating that a minimum temperature and reaction time are necessary to enable effective solvolysis and/or dehydration. In contrast, water and catalyst loading exhibited relatively minor effects on furfural yield.

Optimal conditions were found with the highest catalyst loading and the lowest water loading, which not only confirms the catalytic boosting effect of the POM but also suggests that lower water contents promote the solvolysis and/or dehydration processes more effectively. At these conditions the model predicted a furfural yield of 77.2%, as can be seen in Table S6.† Upon validation a yield of 73.4% was found. The deviation to the model's prediction is likely due to the process conditions lying at the corners of the model for which the Box–Benken-design is not particularly suitable. However, the comparison to a reaction system not containing the HSiW catalyst resulted in lower furfural yields at 67.7%. While this relative increase of approx. 8% with the addition of the POM may not be seen as relevant, the impact on the process economy may be significant, since reaction temperature or time might be reduced while keeping the yield constant resulting in economic benefits and, thus, highlighting the increased efficiency.

Building on the optimized reaction conditions, the reaction kinetics were studied to determine the effective rate constant and activation energy. A pseudo-first-order kinetic model was assumed, based on the hyperstoichiometric availability of dissociated protons, and the initial substrate concentrations were varied. Subsequently, the temperature was adjusted while maintaining all other conditions constant. The results are presented in the double-logarithmic plots shown in Fig. 2.

**Fig. 2:**
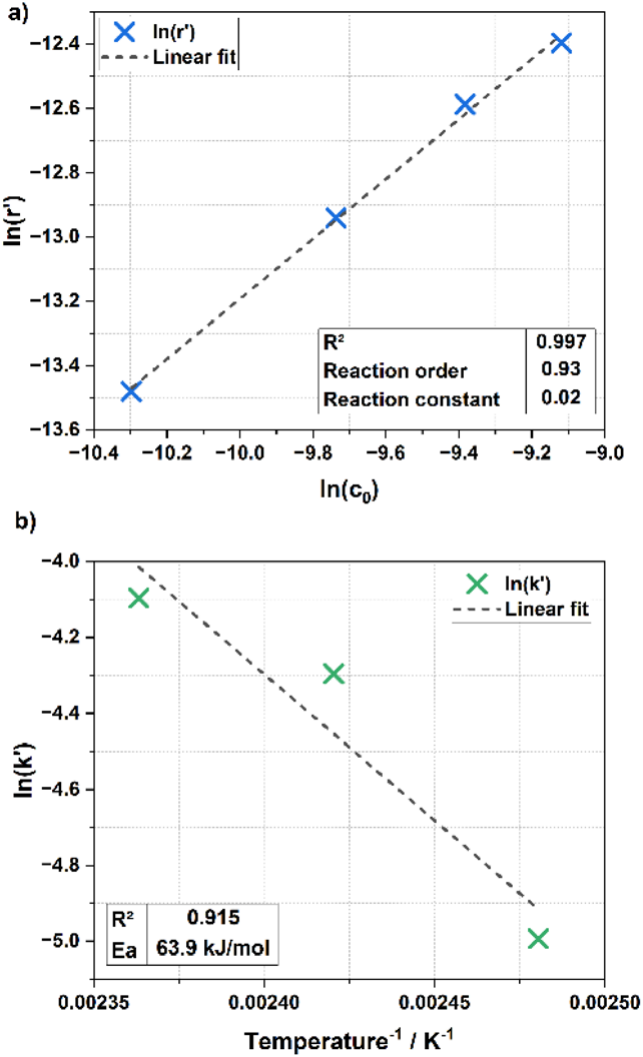
Determination of reaction order (a) and activation energy (b). Reaction conditions (a): 0.1; 0.175; 0.25; 0.325 g (b) 0.25 g sugarcane bagasse, 5 g [DMBA][HSO_4_] (including 20 wt% H_2_O), 0.20 g H_4_SiW_12_O_40_ (HSiW), *t* = 90 min, *T* = (a) 150 °C (b) 130; 140; 150 °C.

As seen in Fig. 2a, the reaction order was determined to be 0.93, confirming the assumption of a pseudo-first-order reaction. The effective rate constant was found to be *k*′ = 0.02 min^−1^, which is on the higher end compared to values reported in other studies.^32,33^ The Arrhenius plot regression in Fig. 2b exhibits higher error than that in Fig. 2a, but the calculated activation energy was approximately *E*_a_ ≈ 64 kJ mol^−1^, which is on the lower end compared to other studies.^34,35^ Despite the moderate error in the regression, these results highlight a significant beneficial effect of the utilized IL and POM system.

A variation of biomass feedstocks (Table S2†), including miscanthus, beech wood, and spruce wood, was tested under optimized reaction conditions, alongside sugarcane bagasse. The results, shown in Table 3, provide insight into the efficiency of furfural production, normalized to hemicellulose content, allowing for a direct comparison. Sugarcane bagasse yields the highest efficiency (73.4%), followed by beech wood (61.4%) and spruce wood (60.9%), while miscanthus shows the lowest (56.1%). According to Yemiş and Mazza,^36^ these variations are due to differences in hemicellulose composition, structural accessibility, and other biomass components like lignin and cellulose. Their study highlights that reaction conditions are critical, as xylose conversion to furfural depends on feedstock characteristics. The higher yield from sugarcane bagasse suggests more accessible hemicellulose, while the lower yield from miscanthus may result from inhibitory compounds or less favourable hemicellulose properties. These findings stress the importance of both the quantity and quality of hemicellulose for optimizing furfural production.

**Table 3:** Furfural yields based on pentosan content with varying lignocellulosic feedstocks at optimized operating conditions. Reaction conditions: 0.25 g sugarcane bagasse, 5 g [DMBA][HSO_4_] (including 20 wt% H_2_O), 0.20 g H_4_SiW_12_O_40_ (HSiW), *t* = 90 min, *T* = 150 °C

Entry	Biomass feedstock	Furfural yield (%)
1	Sugarcane bagasse	73.4
2	Miscanthus	56.1
3	Beech wood	61.4
4	Spruce wood	60.9

In conclusion, this study demonstrated the potential of ionic liquids (ILs) and polyoxometalates (POMs) as an efficient catalytic system for furfural production under mild conditions. The optimization of reaction parameters revealed that temperature and reaction time play the most significant roles in enhancing furfural yield, with the highest efficiency achieved at 150 °C and 90 minutes. Additionally, a variety of lignocellulosic biomass feedstock was tested, showing that sugarcane bagasse yields the highest furfural efficiency, followed by beech wood and spruce wood. The findings underscore the importance of both feedstock quality and reaction conditions in optimizing furfural production, suggesting that the combination of ILs and POMs offers a promising pathway for more sustainable and efficient chemical production.

Perspectively, not only further process and yield optimization should be conducted. Especially the robustness and recyclability of the catalytic system would need to be further investigated, as only IL or POM individually have been studied on this matter, so far.^31,37^ Additionally, the separation or isolation of furfural and other quantified side products shall be investigated by either liquid–liquid-extraction^38^ or distillation. The results of these further investigations could then be applied to a modified techno-economic assessment of the previously published ionoSolv process further promoting the commercialization of POM IL systems.^37^

## Data availability

Data will be made available on reasonable request by the corresponding author.

## Author contributions

Max P. Papajewski performed conceptualization of the project idea, formal analysis for the design of experiment and kinetic study, performing the experiments, analysing products and overall evaluation, and writing the original draft. Suhaib Nisar performed investigation by biomass characterization. Chaoyue Zhang performed investigation by biomass screening experiments, and biomass characterization. Jason P. Hallett provided resources by infrastructure, materials, and laboratory equipment, project supervision, and reviewing & editing the draft. Jakob Albert provided resources by materials, project supervision and reviewing & editing the original draft.

## Conflicts of interest

There are no conflicts to declare.

## Supplementary Material

RA-015-D5RA02401C-s001
