# Supplementary material for: Boosting furfural production by combining polyoxometalates and ionic liquids for effective fractionation of lignocellulosic biomass
Source: RSC Adv. 2025 May 2;15(18):14259–63. doi: 10.1039/d5ra02401c (PMC12046310; doi:10.1039/d5ra02401c)
Supplement: RA-015-D5RA02401C-s001 [file RA-015-D5RA02401C-s001.pdf]

Supplementary Information (SI) for RSC Advances.
This journal is © The Royal Society of Chemistry 2025

Boosting furfural production by combining polyoxometalates and ionic liquids for effective fractionation of lignocellulosic biomass

Max Papajewski[1], Suhaib Nisar[2], Chaoyue Zhang[2], Jason P. Hallett[2], Jakob Albert[1]

[1] Institute for Technical and Macromolecular Chemistry, Universität Hamburg, Bundesstraße 45, 20146 Hamburg, Germany

[2] Department of Chemistry, Imperial College London, South Kensington Campus, London, UK

Contents

1. General experimental Information 2
   1.1 Materials 2
   1.2 Syntheses 2
   1.3 Experimental setup & work procedure 5
   1.4 Analytical Methods 6
2. Experimental Data 8
3. References 10

1. General experimental Information

1.1 Materials

Biomass:

- Sugarcane bagasse was acquired from São João Mill in Araras-SP, Brazil
- Beech wood was purchased from the Mushroom & Equipment Shop
- Spruce wood was acquired from the MORE research facility in Lulea, Sweden
- Miscanthus was acquired from the Silwood Park Campus at Imperial College London, UK

Other chemicals:

The polyoxometalates with the formula $H_3PMo_{12}O_{40}$ (HPMo), $H_3PW_{12}O_{40}$ (HPW), and $H_4SiW_{12}O_{40}$ (HSiW) were purchased from Sigma Aldrich. The Ionic liquids [Hmim][Cl], [Bmim][Cl], [Emim][Et_2PO_4], and [TBMP][$MeSO_4$] were purchased from iolitec, while [Emim][Cl] was purchased from Sigma Aldrich. Deionized water was used from the laboratory facilities. The furfural with a purity of 99.8 % used for calibration was purchased from Sigma Aldrich. A chromatogram of the furfural utilized for calibration showing its purity can be seen in Figure S5. For the determination of acid-base-ratio, sodium hydroxide was utilized and previously purchased from Sigma Aldrich. For the determination of water content by Karl-Fischer-titration, Honeywell Hydranal Methanol, Composite and calibration standards were utilized.

1.2 Syntheses

Polyoxometalates:

The identity of the $H_4SiMo_{12}O_{40}$ (HSiMo) catalyst was synthesized according to *Strickland* (1952)[1]. MoO_3 (34.55 g) was suspended in H_2O (500 mL), and NaOH (12.75 g) was added until complete dissolution of the solid. A solution of Na_2SiO_3 (2.44 g) in a small amount of H_2O was then added to the molybdate under vigorous stirring, yielding a yellow solution. This solution was acidified with HCl (1 mol/L) to pH 1.4. An attempt to extract the product from the acidic solution using diethyl ether was unsuccessful. Further acidification to pH 0.745 was carried out, but the organic phase remained colorless. Both organic and aqueous phases were concentrated, yielding 10.00 g of crude product.

The crude product was dissolved in 100 mL of water, forming a greenish-yellow solution with some white solid precipitating at the bottom. The addition of 10 mL HCl (37%) and 10 mL H_2O_2 (35%) turned the solution bright yellow, but the white solid remained undissolved and was subsequently filtered off. The filtrate was extracted with 10 × 50 mL $C_4H_8O_2$, resulting in an intensely yellow organic phase, while the aqueous phase retained most of its color. Finally, the organic phase was concentrated to dryness, yielding an amorphous green solid resulting in the elemental composition shown in Table S1. Measured by ICP-OES and F-AAS.

This specific batch was also utilized and characterized by Wesner et al. Further analytical characterization can be seen in the corresponding ESI.[2]

Table S1: Elemental composition and stoichiometric ratio of the $H_4SiMo_{12}O_{40}$ (HSiMo) POM catalyst.

**Element**	Na	Si	Mo
**Mass fraction / wt.-%**	0.46	1.52	54.33

**Stoichiometric ratio**	0.42	1.15	12
**Targeted ratio**	0	1	12

Ionic liquids

The ionic liquid [DMBA][HSO_4] was synthesized according to *Geschwend* (2016).[3] 5 M sulphuric acid (500 mL, 2.5 mol) was added to *N*,*N*-dimethyl-*N*-butylamine (252.80 g, 2.5 mol) being in an ice-bath. The mixture was stirred for 3 hours. Excess of water was evaporated, and a transparent viscous liquid was obtained (499.00 g, 2.5 mol, 100%).

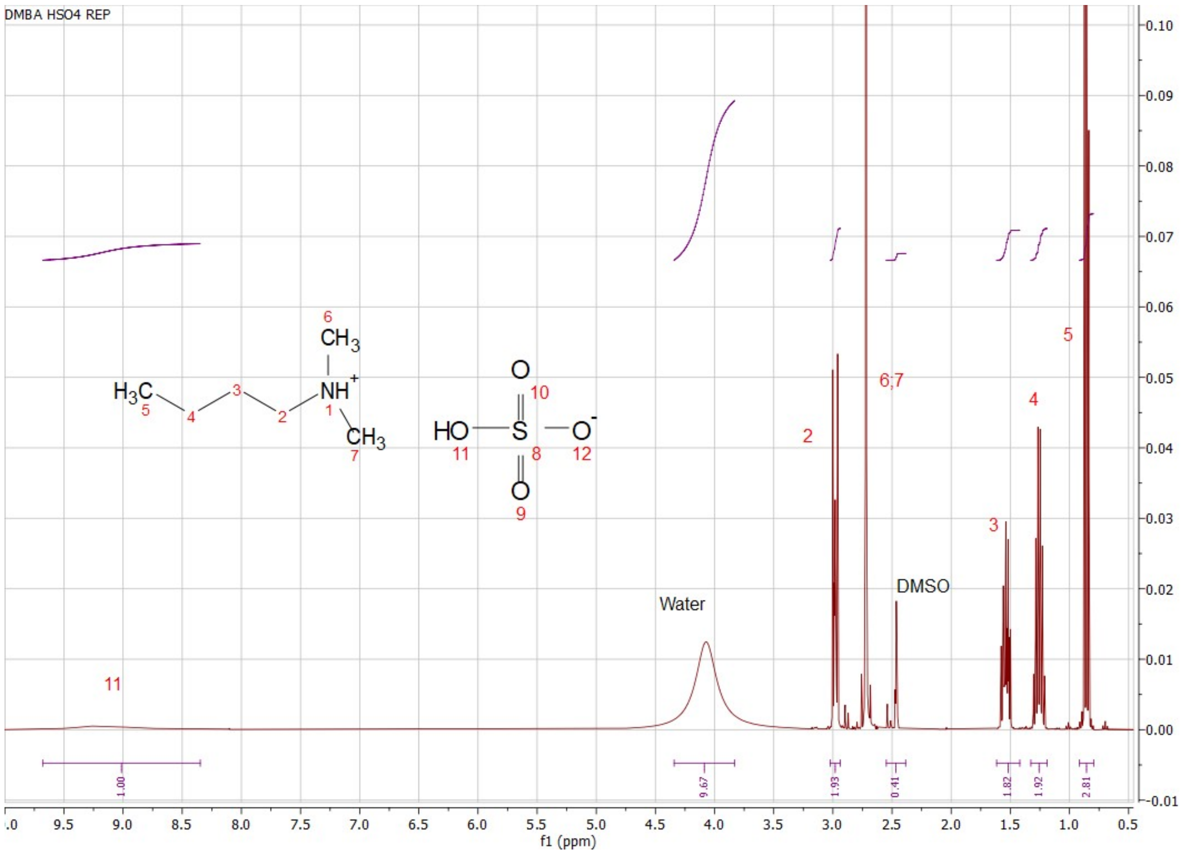

Figure S1: ^{1}H-NMR of [DMBA][HSO4] ionic liquid.

1.3 Experimental setup & work procedure

The experiments conducted were all carried out by following the laboratory standard operating procedure of *Hallett* research group.[3] A schematic representation of the reaction procedure is depicted Figure S1. The ionic liquid/water solvent system, the polyoxometalate and the biomass were weighed in an Ace Pressure Tube utilizing an analytical balance (Satorius with a precision of ±0.0001 g) and tightly sealed with provided sealings and lids.

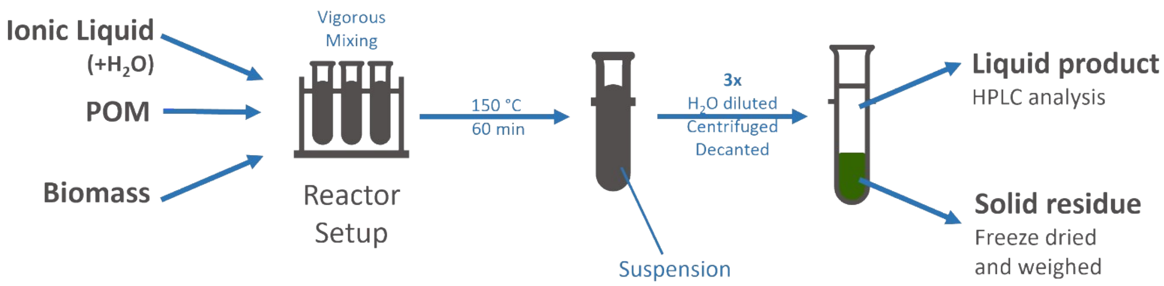

Figure S2: Schematic representation of the reactor setup.

For a better understanding, a depiction of four sealed pressure tubes containing ionic liquid, polyoxometalate and biomass prior to mixing are shown in Figure S2A. As can be seen, the contents of the pressure tube were heterogeneous and required mixing, at this stage. This was done by holding each pressure tube on a vortex mixer for a couple of seconds ensuring no solids adhered to the tube walls.

(A) (B)

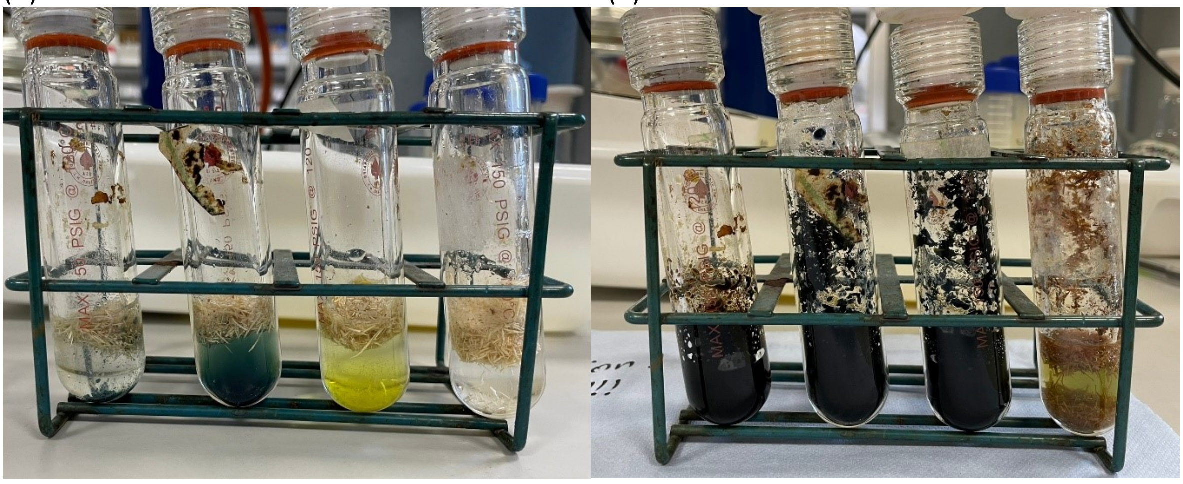

Figure S3: Depiction of pressure glass tubes containing all reactants before (A) and after reaction (B).

The pressure tubes were then again secured in a rack and placed into a preheated oven at the designated reaction temperature (between 110 and 150 °C). Reaction time commenced immediately and was terminated after either 30, 60, or 90 min by transferring the rack containing the pressure tubes to a fume hood for cooling. Once the pressure tubes reached room temperature approx. after

30 min, each tube was opened, and all contents were transferred to pre-weighed centrifuge-applicable containers. Any adhering contents on the pressure tube's wall were rinsed with deionized water. The centrifuge container was then filled with deionized water causing lignin to precipitate. The containers were closed and again thoroughly mixed by a vortex mixer before being placed in a centrifuge which was run at 3,500 rpm for 20 min. The liquid sample in the container was then decanted into a pre-weighed liquid sample storage bottle. This procedure of filling, mixing, centrifuging and decanting the centrifuge container was repeated two more times to ensure complete precipitation of lignin and other residues (solid residue), to solubilize the polyoxometalate allowing for further characterization of the solid residue, and to dilute the liquid sample allowing for better furfural quantification.

The storage bottle containing the liquid sample of all three decanting steps was again weighed to gain the total liquid product weight. Using a syringe and a syringe filter (<0.2 µm) some of the liquid product was transferred to an HPLC vial which was then measured on the Shimadzu HPLC system for furfural quantification.

The solid residue was freeze dried by previously freezing it with liquid nitrogen and then placing it in a Labconco Freezone 6 freeze drier operating at <0.03 mbar and -40 °C. After drying for at least 24 h, the container was weighed gaining the solid residue mass.

1.4 Analytical Methods

Compositional analysis:

The compositional analysis was conducted according to NREL protocol.[4] Each biomass sample was analyzed for acid-insoluble and acid-soluble lignin, ash, and sugar contents in triplicates. The equipment used included an analytical balance (Satorius with a precision of ±0.0001 g), oven (VWR Venti-Line 115), muffle oven (Nabertherm P330), autoclave (Sanyo Labo ML5 3020U), pH meter (VWR SB70P), and UV-Vis spectrometer (Perkin Elmer Lambda 650).

Air-dried pulp (300 mg, oven-dry basis) was weighed into a 100 mL Ace pressure tube, mixed with 3 mL of 72% sulfuric acid, and stirred every 15 minutes for 1 hour in a 30 °C water bath. The mixture was diluted with 84 mL distilled water, autoclaved (120 °C, 1 h), cooled (80 °C, 1 h), and filtered through a pre-weighed ceramic crucible. The filtrate was collected for UV and HPLC analysis.

The solid residue was washed with hot distilled water, dried at 105 °C overnight, weighed, then ashed at 575 °C in a muffle oven. The masses of the empty crucible, residue, and ash were recorded to calculate acid-insoluble lignin (AIL) and ash contents using Equations 1 and 2, where m_0 is the mass of the empty crucible, $m_{AIL + Ash}$ is the mass of the crucible with acid insoluble lignin and ash, m_{Ash} is the mass of the crucible with ash, and $m_{oven\ dried\ pulp}$ is the mass of the sample (approx. 300 mg)

$$AIL(\%) = \frac{(m_{AIL + Ash} - m_0) - (m_{Ash} - m_0)}{m_{oven\ dried\ pulp}} \times 100\% \qquad \text{Eq.1}$$

$$Ash(\%) = \frac{m_{Ash} - m_0}{m_{oven\ dried\ pulp}} \times 100\% \qquad \text{Eq.2}$$

Filtrate samples were analyzed via UV-Vis at 240 nm. The ASL content was determined using Equation 3, incorporating UV absorbance, filtrate volume, sample mass, absorptivity (ε), cuvette path length (l), and dilution factor (d).

$$ASL(\%) = \frac{UV_{avg.\,Absorbance} \times mVolume_{Filtrate}}{m_{oven\,dried\,pulp} \times \epsilon \times l \times d} \times 100\%$$
Eq.3

Sugars (glucose, xylose, arabinose, galactose, mannose) were quantified using an HPLC system (Shimadzu) with an AMINEX HPX-87P column (BioRad) and RI detector. The mobile phase was purified water (18 MΩ) at 0.6 mL/min, with a column temperature of 85 °C and a runtime of 20 min. Calibration standards (0.1–4 mg/mL for all sugars; additionally, 8 mg/mL for glucose) were used. Sugar content was calculated via Equation 4, where c_{HPLC} is the sugar concentration recorded by HPLC, V is the initial sample volume, $corr_{anhydro}$ is the correction for the mass increase during polymeric sugars hydrolysis, and SRC is the sugar recovery coefficient.

$$Sugar(\%) = \frac{c_{HPLC} \times V \times corr_{anhydro}}{m_{oven\,dried\,pulp} \times SRC} \times 100\%$$
Eq.4

Standards were prepared in 10 mL aqueous solutions, adjusted with sulfuric acid, sealed, and autoclaved. The sugar recovery coefficient was determined as per Equation 5.

$$SRC = \frac{c_{HPLC} \times V \times corr_{anhydro}}{m_{oven\,dried\,pulp} \times SRC} \times 100\%$$
Eq.5

The results of the compositional analysis of the biomass samples are shown in Table S1.

Table S2: Results of compositional analysis for all utilized biomass feedstocks in this study.

	**Sugarcane Bagasse**	**Miscanthus**	**Spruce wood**	**Beech wood**
**Glucan**	40.23%	49.46%	43.37%	42.02%
**Xylan**	21.68%	21.28%	5.54%	17.47%
**Galactan**	0.00%	0.00%	2.38%	0.00%
**Arabinan**	2.11%	1.75%	1.03%	0.00%
**Mannan**	0.12%	0.00%	12.25%	0.51%
**Acid soluble lignin**	4.58%	3.33%	1.49%	9.79%
**Acid insoluble lignin**	25.47%	20.90%	25.79%	25.09%
**Ash**	0.62%	0.23%	0.03%	0.27%
**Extractives**	5.19%	3.05%	8.11%	4.85%

Acid-base-ratio

The acid-base-ratio was measured for the ionic liquid [DMBA][HSO4] utilizing a Mettler Toledo Compact Titrator G20S. Triplicate measurement was conducted to ensure the acid-base-ratio of the ionic liquid is close to a value of 1. In Table S2 the results of the measurements are shown confirming a balance acid-base-ratio. The water content of the ionic liquid was 19.99 %, the molecular weight of the acid, the amine and the total ionic liquid (IL) are 98.08, 101.19, and 199.27 g/mol, respectively.

Table S3: Results of acid-base-ratio measurements for the primarily utilized ionic liquid [DMBA][HSO4].

IL mass (g)	IL mass dry basis	mmoles of NaOH consumed	Moles of NaOH consumed	Moles of HSO_4	Acid/Base ratio
0.1767	0.141	0.684	0.00068400	0.000684000	0.976
0.0959	0.077	0.39	0.00039000	0.000390000	1.009
0.0733	0.059	0.29	0.00029000	0.000290000	0.990

Karl-Fischer titration

For the determination of water content, a Mettler Toledo V20 Volumetric Karl-Fischer titrator was utilized in combination with Honeywell Hydranal™ compounds for calibration and measurement. The self-synthesized ionic liquid [DMBA][HSO_4] was measured in triplicate according to the instructions issued by the titrator manufacturer. A few drops of IL are added to the titrator using a pre-weighed syringe. The weight of the added sample was entered and the measured water content displayed was recorded leading to the average water content of $Water(\%) = 19.99\ wt.-\%$ for [DMBA][HSO_4].

HPLC for furfural quantification

For the quantification of furfural in the product samples a Shimadzu HPLC system equipped with an Agilent Eclipse XDB-C18 column at a flow rate of 0.6 mL/min, a temperature of 55 °C and a 8:2 v/v water/methanol mobile phase was utilized. The furfural yields were calculated according to Equation 6, where $c_{Furfural}$ is the measured concentration of furfural in mg/mL, V_{Sample} is the volume of the sample acquired by the mass of the sample and an assumed density of water as the sample is highly diluted as described in the experimental procedure, $m_{Biomass}$ is the initially weighed biomass, w_{dried} is the dried fraction of the biomass, $w_{Hemicellulose}$ is the fraction of hemicellulose based on a dried biomass, and M is the molar mass of the respective indexed component.

$$Y_{Furfural} = \frac{c_{Furfural} \cdot V_{Sample}}{m_{Biomass} \cdot w_{dried} \cdot w_{Hemicellulose}} \cdot \frac{M_{Xylose} - M_{Water}}{M_{Furfural}} \qquad \text{Eq.6}$$

2. Experimental Data

Design of Experiment study

Table S4: Parameter and level selection for full-factorial three-level design-of-experiment study.

Factor	Level 1	Level 2	Level 3	Unit
**Temperature in °C**	110	130	150	°C
**Reaction time in min**	30	60	90	min
**Water loading in wt.-%**	20	40	60	wt.-%
**Catalyst loading in wt.-%**	1	2.5	4	wt.-%

Table S5: Parameter and level selection for full-factorial three-level design-of-experiment study.

**Entry**	**Temperature in °C**	**Reaction time in min**	**Water loading in wt.-%**	**Catalyst loading in wt.-%**	**Furfural yield in mole-%**
1	110	30	40	2.5	0%
2	110	60	20	2.5	0.19%
3	110	60	40	1	0%
4	110	60	40	4	0%
5	110	60	60	2.5	0%
6	110	90	40	2.5	0.66%
7	130	30	20	2.5	0.29%
8	130	30	40	1	1.88%
9	130	30	40	4	0.03%
10	130	30	60	2.5	0.23%
11	130	60	20	1	13.93%
12	130	60	20	4	4.29%
13	130	60	40	2.5	6.78%
14	130	60	40	2.5	7.87%
15	130	60	40	2.5	7.66%
16	130	60	60	1	17.94%
17	130	60	60	4	5.11%
18	130	90	20	2.5	12.35%
19	130	90	40	1	26.99%
20	130	90	40	4	9.38%
21	130	90	60	2.5	15.66%
22	150	30	40	2.5	14.26%
23	150	60	20	2.5	52.89%
24	150	60	40	1	40.00%
25	150	60	40	4	32.70%
26	150	60	60	2.5	27.88%
27	150	90	40	2.5	55.59%

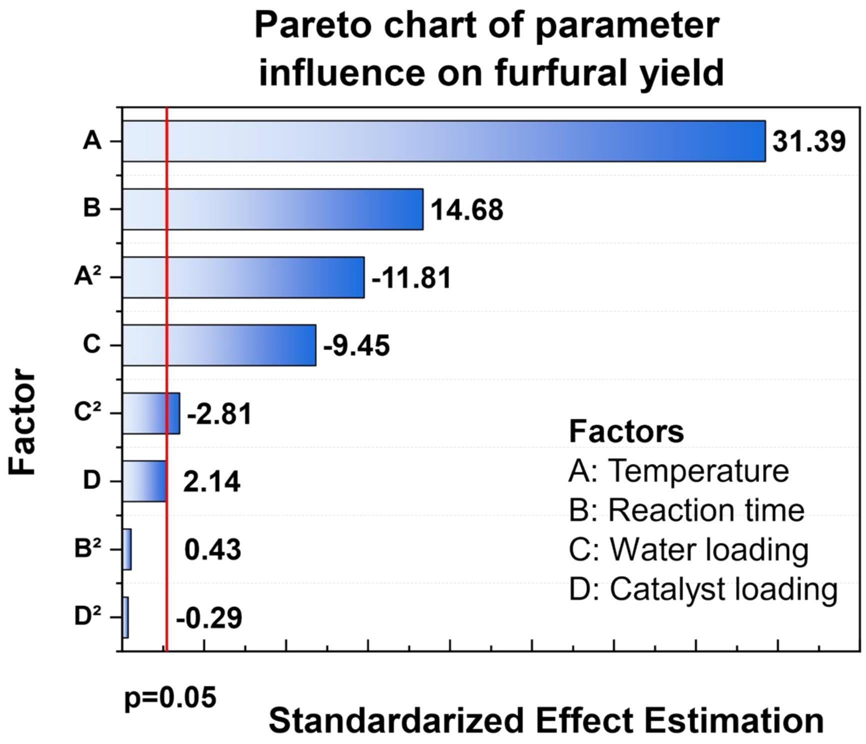

Figure S4: Pareto chart of the standardized effect estimation of selected parameters on furfural yield (excluding factor-interactions).

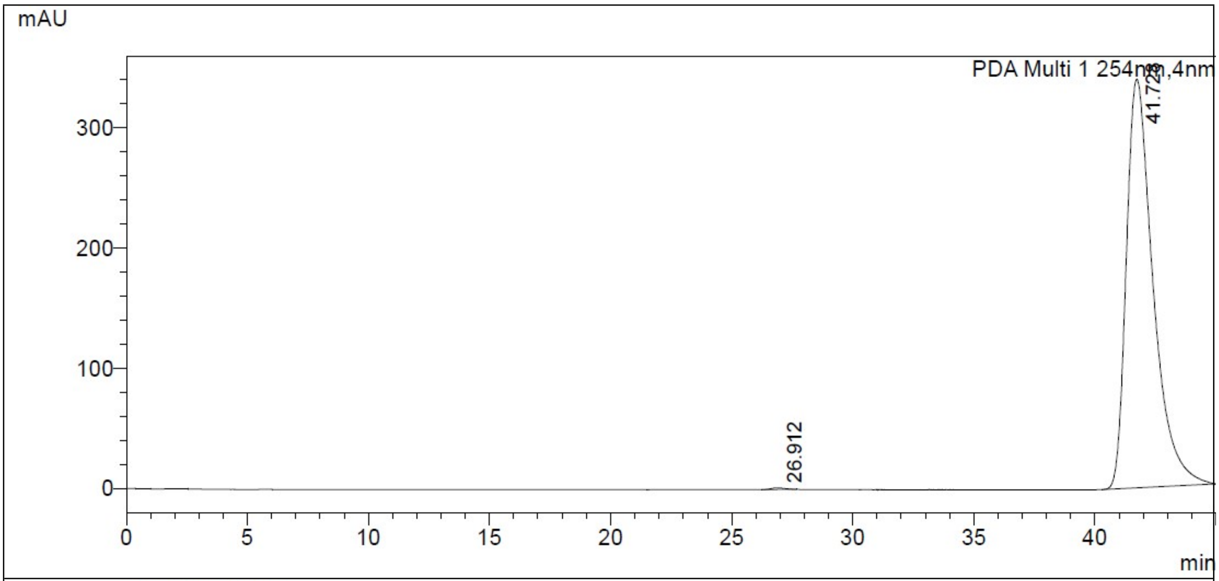

PDA Ch1 254nm

Peak#	Ret. Time	Area	Height	Conc.	Unit	Mark	Name
1	26.912	55399	1282	-0.019	mg/L		furoic acid
2	41.728	26096457	340167	0.519	mg/L		furfural
Total		26151856	341449				

Figure S5: Chromatogram of furfural utilized for calibration.

Table S6: Validation of DoE parameter optimization in comparison to pure ionic liquid system. Substrate: Sugarcane bagasse, ionic liquid: Dimethylbutylamine hydrogen sulphate ([DMBA][HSO4]), additive: H4SiW12O40 (HSiW) or none, substrate mass: 0.250 g, ionic liquid mass: 5 g, water loading: 20 wt.-%, catalyst loading: 4 % (200 g), reaction time: 90 min, temperature: 150 °C.

Catalytic system	Description	Furfural yield (mol-%)
[DMBA][HSO_4] + HSiW	Validation of prediction	73.4
[DMBA][HSO_4] + HSiW	Predicted value	77.2
[DMBA][HSO_4]	Comparison with no HSiW catalyst	67.7

3. References

1 J. D. H. Strickland, *J. Am. Chem. Soc.*, 1952, **74**, 862–867.

2 A. Wesner, et al., *RSC Adv.*, 2025, **15**, 38–47.

3 F. J. V. Gschwend, et al., *JoVE*, 2016. DOI: 10.3791/54246-v.

4 A. Sluiter, et al., *Determination of Structural Carbohydrates and Lignin in Biomass. Laboratory Analytical Procedure*, Golden, CO, 2012.
